# Simultaneous Multiple Thromboembolic Events in a Postpartum Patient

**DOI:** 10.5811/cpcem.2018.5.37451

**Published:** 2018-06-16

**Authors:** Ibtisam Ghashimi, Linda Jafarah, Amal Bakhsh, Ahmed Azzazy, Ahmed Royashed, Awad Awad, Ahmed Ramadan, Ghofran Hashimi, Refah Alqahtani, Abdullah Bakhsh

**Affiliations:** * Ibn Sina National College of Medicine, Emergency Medicine, Jeddah, Saudi Arabia; † King Abdulaziz University, Emergency Medicine, Jeddah, Saudi Arabia; ‡ King Abdulaziz University, Intensive Care Unit, Jeddah, Saudi Arabia; § King Abdulaziz University, Radiology, Jeddah, Saudi Arabia; ¶ King Abdulaziz General Hospital, Radiology, Jeddah, Saudi Arabia

## Abstract

We report the case of a postpartum patient who presented to the emergency department in status epilepticus. She was initially treated for eclampsia; however, she was subsequently found to have simultaneous cerebral venous thrombosis (CVT) and pulmonary embolism (PE). While thromboembolic events may be seen frequently in the postpartum period, the combination of CVT and PE is an unusual occurrence. Although a challenging diagnosis, the emergency physicians played a critical role in the early recognition and rapid treatment of CVT in this case.

## INTRODUCTION

Cerebral venous thrombosis (CVT) is an unusual but devastating neurological emergency.^1^ It accounts for 0.5%–1% of all strokes in the general population.^2^ The incidence increases during pregnancy and in the postpartum period, accounting for 6%–64% of pregnancy-related strokes.^3^ Nonetheless, CVT remains a diagnostic challenge in the emergency department (ED) due to the remarkably wide spectrum of signs and symptoms. In addition, magnetic resonance imaging (MRI) as a screening modality is not easily accessible in the ED.

## CASE REPORT

An 18-year-old female who was 10 days post-vaginal delivery presented to the ED in status epilepticus for which she required endotracheal intubation. She had a blood pressure of 163/89 millimeters of mercury, a heart rate of 155 beats per minute, a temperature of 37.0°Celsius, a respiratory rate of 22 breaths per minute, and an oxygen saturation of 94% on 15L per minute of oxygen via a bag-valve mask. Physical examination confirmed the presence of left leg swelling with mild erythema below the knee; otherwise, no palpable cords or other abnormalities were seen in her lower extremities. Initially it was thought to be deep venous thrombosis, but there was no evidence via venous Doppler ultrasound. Cardiac examination did not reveal murmurs, rubs, gallops, or other abnormalities, and her lungs were clear to auscultation. Upon questioning her family, it was revealed that a few hours prior to presentation the patient had developed sudden onset of difficulty breathing and subsequent loss of consciousness. She was rushed to the ED.

Further workup revealed leukocytosis of 19.84×10^9^ /L, elevated D-dimer of 19.9 milligrams per liter, fibrinogen of 457.9 milligrams per deciliter, and troponin of 2.42 micrograms per liter. An electrocardiogram (EKG) revealed an S1Q3T3 pattern. A urine dipstick revealed +2 protein and was otherwise normal.

**Image 1:**
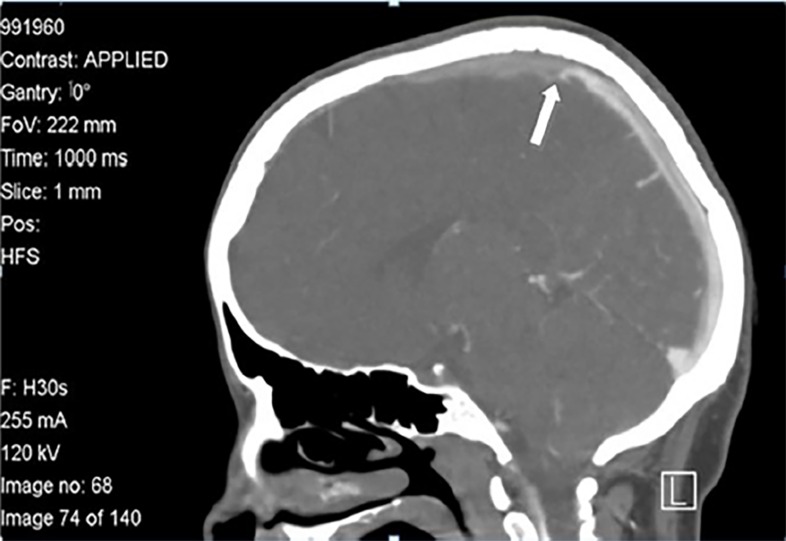
Sagittal computed tomography venogram of the brain showing filling defects involving most of the superior sagittal sinus (arrow), except the posterior portion, suggesting superior sagittal sinus thrombosis.

**Image 2:**
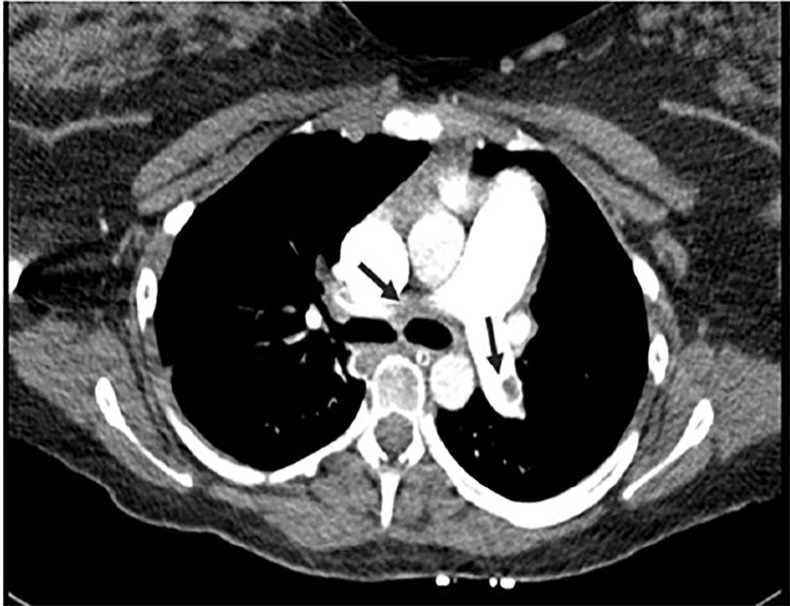
Computed tomography pulmonary angiogram showing bilateral large filling defects involving the main pulmonary arteries (arrows).

A magnesium sulfate (MGSO4) bolus dose of 4g intravenous (IV) over 30 minutes followed by a drip of 2 grams per hour was initiated for presumed eclampsia. A brain computed tomography (CT) without contrast was ordered for the workup of a first-time seizure. This was unremarkable. At this point, the patient’s differential diagnosis was reconsidered and prompted the team to order a CT venogram (CTV) of the brain with a CT pulmonary angiogram. It demonstrated CVT involving the superior sagittal and right transverse sinuses (Image 1), while her CT pulmonary angiogram showed bilateral pulmonary thromboses involving the main, lobar, and multiple segmental arteries bilaterally (Image 2).

**CPC-EM Capsule**

What do we already know about this clinical entity?

Cerebral venous thrombosis is a devastating neurological emergency. The incidence during pregnancy and postpartum period, account for 6% to 64% of pregnancy-related strokes.

What makes this presentation of disease reportable?

Although this case was not completely novel, pulmonary embolism and cerebral venous sinus thrombosis (CVST) are not reported in the literature as presenting simultaneously.

What is the major learning point?

CVST is a challenging diagnosis, and this case reinforces the reminder to consider it when evaluating syncope, seizure, headache, or any neurological complaint.

How might this improve emergency medicine practice?

This case is an important reminder to include CVST in there differentials when approaching postpartum patients with seizure or any neurological sequel.

**Image 3:**
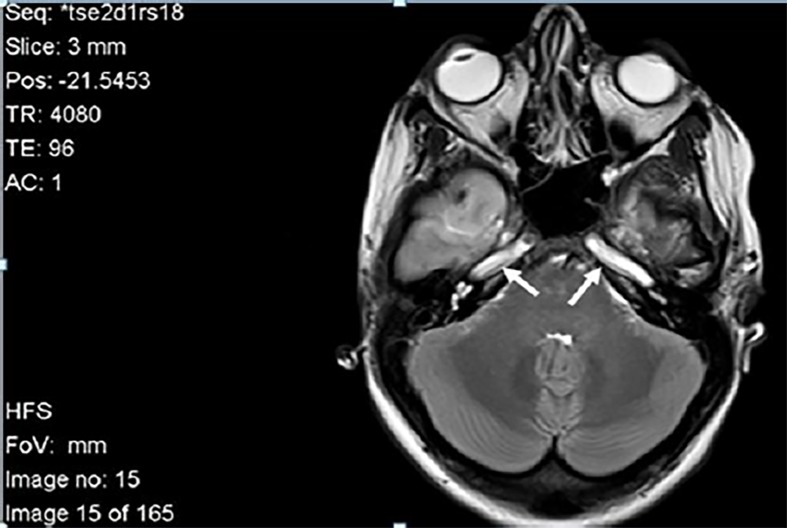
Magnetic resonance angiogram showing absent signal flow in both internal carotid arteries (arrows), indicating occlusion.

Enoxaparin sodium was initiated in the ED and the patient was admitted to the intensive care unit (ICU) for further management. During her ICU stay, brain MRI with a magnetic resonance angiogram showed diffuse ischemia with a 5 mm tonsillar herniation and the absence of signal flow void in both internal carotid arteries (Image 3). She was started on 1 g/kg IV of mannitol over 60 minutes. Additionally, a presumptive diagnosis of seronegative antiphospholipid syndrome was made and IV immunoglobulin and methylprednisolone were initiated, with the addition of plasmapheresis. Unfortunately, none of the treatment measures showed a favorable response. Her poor prognosis was discussed with her family and the consensus was to place a do-not-resuscitate order. The patient died in the ICU two weeks later because of tonsillar herniation.

## DISCUSSION

CVT is a major cause of morbidity and mortality if left unrecognized.^4^ Setbacks in the diagnosis are due to the infrequency of the disease combined with variability in manifestations and lack of specialized imaging in the ED.^4^ Patient outcomes can be enhanced with prompt recognition and treatment. Generalized tonic-clonic seizures are present in 40% of those with CVT, as was the case in our patient.^1^ Therefore, it is prudent for emergency physicians to include a CVT in the differential diagnosis of seizing patients, especially in all hypercoagulable states. This will improve recognition, allow the early initiation of treatment, and reduce morbidity and mortality. Other initial findings include headache (81%), limb weakness (34%), disturbed consciousness (30%), blurred vision (11%), and fever (6.9%).^1^

Head CT without contrast is the first-line investigation in patients with new headache, focal neurologic deficit, seizure, or altered mental status as per the guidelines of the American College of Emergency Physicians.^5^ Unfortunately, this test has a poor sensitivity for CVT and is normal in 30% of cases.^6^ Therefore, a normal non-contrasted CT of the brain should not be used alone to rule out the diagnosis of CVT.^7^ However, CT combined with venography is reliable with an overall sensitivity of 95%,^6^ which is comparable to the sensitivity of magnetic resonance venography. The widespread availability of CTV makes it the initial test of choice for the diagnosis of CVT.^6^ A normal D-dimer level may be considered to help identify patients with low probability of CVT. However, if there is a strong suspicion of CVT, a normal D-dimer level should not preclude further evaluation.^6^

In our patient, it is likely that a pulmonary embolism (PE) might have caused her CVT, as her respiratory symptoms appeared earlier than her neurological symptoms. The association of CVT and PE may be explained by a detached thrombus and global prothrombotic state.^8^

Mortality due to CVT can reach 5.6% during the acute phase, commonly due to herniation.^9^–^11^ Postpartum women have a higher risk for both venous thromboembolism and eclampsia. Although this case was not completely novel, PE and cerebral venous sinus thrombosis (CVST) are not reported in the literature as presenting simultaneously, and our case was unique in that the patient’s presentation was initially consistent with eclampsia (hypertension, syncope, seizure, and mild proteinuria). CVST is a challenging diagnosis, and this case reinforces the reminder to consider it when evaluating syncope, seizure, headache, or any neurological complaint. This case is applicable to emergency medicine providers to improve the survival rates of these patients.

## CONCLUSION

Cerebral venous thrombosis can be commonly missed in the ED. It is critical for emergency physicians to include CVT in the differential diagnosis of patients in the ED and to proceed with appropriate imaging if suspicion is high. Venous thromboembolism remains the most common cause of direct maternal deaths, and morbidity and mortality are significantly reduced with improvements in recognition and treatment options.

Documented patient informed consent and/or Institutional Review Board approval has been obtained and filed for publication of this case report.
